# FUNCTIONAL CHARACTERIZATION OF ENDOCANNABINOID RELATED 2-AG SYNTHASES IN DOPAMINERGIC NEURONS

**DOI:** 10.1093/geroni/igae098.3661

**Published:** 2024-12-31

**Authors:** Quentin Price, Disa Basu, Huaibin Cai

**Affiliations:** Howard University, Los Angeles, California, United States; National Institute of Aging, Bethesda, Maryland, United States; National Institute of Aging, Bethesda, Maryland, United States

## Abstract

We previously linked several loss of function mutations of the enzyme diglycerol lipase beta (DAGLB) to early onset Parkinsonism. Parkinson’s disease is defined as the loss of dopaminergic neruons (DANs) in the substantia nigra compacta (SNC). DAGLB is the main synthase of 2-arachidonyl glycerol (2-AG) in nigral DANs. 2-AG from the nigral DANs disinhibits presynaptic inputs such as the GABAergic inputs from the direct spiny projection neurons (dSPNs) of the striatum. However, DAGLA is expressed in nigral DANs as well. We asked ourselves if decreasing the expression fo both DAGLA and DAGLB in the nigral DANs of mice induce motor phenotypes of Parkinson’s disease.

